# Supplementary material for: Dynamic similarity and the peculiar allometry of maximum running speed
Source: Nat Commun. 2024 Mar 11;15:2181. doi: 10.1038/s41467-024-46269-w (PMC10928110; doi:10.1038/s41467-024-46269-w)
Supplement: Supplementary file 1 — Supplementary Information [file 41467_2024_46269_MOESM1_ESM.pdf]

Dynamic similarity and the peculiar allometry of maximum running speed

David Labonte[1], Peter J Bishop[2, 3], Taylor JM Dick[4] & Christofer J Clemente[4, 5]

[1]Department of Bioengineering, Imperial College London, United Kingdom
[2]Museum of Comparative Zoology, Harvard University, USA
[3]Geosciences Program, Queensland Museum, Australia
[4]School of Biomedical Sciences, University of Queensland, Australia
[5]School of Science and Engineering, University of the Sunshine Coast, Australia

Supplementary Note 1

For a Hill-type muscle, the equation of motion may be written as (Labonte, 2023):

$$\begin{aligned} \frac{dv}{dt}d\delta &= \frac{F_{max}G}{m}f(\dot{\varepsilon}_{rel})f(\varepsilon)d\delta \\ \int v\frac{1}{f(\dot{\varepsilon}_{rel})}dv &= \int \frac{F_{max}G}{m}f(\varepsilon)d\delta \end{aligned} \tag{S 1}$$

Here, m is the mass that is moved, assumed to be much larger than the muscle mass, F_{max} is the maximum isometric force, G is the gear ratio, and $f(\dot{\varepsilon}_{rel})$ and $f(\varepsilon)$ are the force-strain rate and force-strain relationship of muscle; $\dot{\varepsilon}_{rel} = \dot{\varepsilon}\dot{\varepsilon}_{max}^{-1}$ is the strain rate normalised with its maximum, and $\varepsilon = \Delta l_m l_m^{-1}$, is the muscle strain.

The advantage of this writing – a re-expression of Newton's second law as a path-integral – is that it enables a symbolic solution via separation of variables of what is otherwise a nonlinear differential equation: the muscle strain, ε, and strain rate, $\dot{\varepsilon}$, are coupled to the displacement, δ, and the speed, v, of the mass via $\delta = l_m\varepsilon G^{-1}$ and $v = l_m\dot{\varepsilon}G^{-1}$.[‡]

For trivial FL and FV relationships, i. e. $f(\varepsilon) = f(\dot{\varepsilon}_{rel}) = 1$, eq. S 1 is the textbook Work-Energy-Theorem for a constant net force. The Hill-limit, v_{Hi}, the Borelli-limit, v_{Bo}, and the physiological similarity index, Γ, then follow from evaluation of each integral for the maximum possible upper integration boundary (Labonte, 2023):

$$\begin{aligned} \int_0^{v_{max}} mv dv &= \int_0^{\delta_{max}} F_{max}G d\delta \\ \frac{1}{2}m\left(\frac{l_m\dot{\varepsilon}_{max}}{G}\right)^2 &= \hat{F}_{max}l_m\varepsilon_{max} \\ \Gamma = \frac{1}{2}\left[\frac{l_m^2}{G^2}\right]\left[\frac{\dot{\varepsilon}_{max}^2}{\hat{\sigma}_{max}\varepsilon_{max}}\right]\left[\frac{m}{V_m}\right] &= \frac{E_{max}}{W_{max}} \end{aligned} \tag{S 2}$$

where $\hat{\sigma}_{max}$ is strictly the average stress exerted as the mass is displaced by δ_{max}, and care is required to correctly account for gearing.

In the main manuscript, we assume that muscle contracts along the plateau of the force-strain relationship, $f(\varepsilon) = 1$, and describe the FV relationship with a linear function, $F_m = F_{max}\left(1 - \frac{\dot{\varepsilon}}{\dot{\varepsilon}_{max}}\right)$ – a special case of the hyperbolic Hill-relation (McMahon, 1984). Whence:

[‡]Provided that in-series elasticity, for example due to tendon-stretch, is absent.

$$\int v \left[\frac{1}{1 - v v_{\mathrm{Hi}}^{-1}} \right] dv = \int \frac{F_{max} G}{m} d\delta \tag{S 3}$$

where we again used $v = l_m \dot{\varepsilon} G^{-1}$ and introduced the Hill-limit, $v_{\mathrm{Hi}} = l_m \dot{\varepsilon}_{max} G^{-1}$.

In order to solve eq. S 3, integration boundaries need to be specified – one limit needs to be fixed, and one is variable, i. e. either the displacement or the velocity are the fixed vs state parameter, respectively. For a muscle which generates a constant force, the magnitude of the effective inertia Γ uniquely determines this choice; for $\Gamma < 1$, the speed is fixed, and for $\Gamma > 1$ the displacement is fixed. For a Hill-muscle, i. e. a muscle which exerts zero force when contracting with the maximum strain rate, the fixed limit is determined instead by a mathematical subtlety: the Hill-relation is asymptotic, i. e. the maximum strain rate is only reached in the limit of an infinitely long contraction. Notationally, a Hill-muscle thus always reaches ε_{max} before it reaches $\dot{\varepsilon}_{max}$, and the integration limit of the path integral is thus always fixed (Labonte, 2023). Evaluation of Eq. S 3 with displacement as the fixed and speed as the system parameter thus yields the correct asymptotic speed limit for any value of Γ. But this is strictly a mathematical and not a physiological or physical result—no muscle contracts for an infinite time, $\dot{\varepsilon}_{max}$ as defined by the Hill-relation is a purely mathematical construct, and the Hill-limit is thus exact only in the unphysical limit of an infinitely long contraction. In practise, this problem may be resolved by defining a cut-off close to $\dot{\varepsilon}_{max}$ as a realistic maximum strain rate, which retains the relevant scalings.

The integral on the right-hand side evaluates to:

$$\int_0^{\delta_{max}} \frac{F_{max} G}{m} d\delta = \frac{F_{max} G}{m} \frac{l_m \varepsilon_{max}}{G} = \frac{1}{2} v_{\mathrm{Bo}}^2 \tag{S 4}$$

The integral on the left-hand side has the closed-form symbolic solution:

$$\int_0^{v_{\mathrm{Hi\text{-}Bo}}} v \left[\frac{1}{1 - v v_{\mathrm{Hi}}^{-1}} \right] dv = - \frac{v_{\mathrm{Hi}}^{-1} v_{\mathrm{Hi\text{-}Bo}} + \log \left[1 - v_{\mathrm{Hi}}^{-1} v_{\mathrm{Hi\text{-}Bo}} \right]}{\left[v_{\mathrm{Hi}}^{-1} \right]^2} \tag{S 5}$$

The Hill-Borelli-limit follows from combination of eqs. S 4 and S 5, solved for $v_{\mathrm{Hi\text{-}Bo}}$:

$$v_{\mathrm{Hi\text{-}Bo}} = v_{\mathrm{Hi}} \left[1 + \mathrm{W} \left(-\exp \left(-1 - \frac{1}{2\Gamma} \right) \right) \right] \tag{S 6}$$

where W is the Lambert W (or ProductLog) function, and we used $\Gamma = \left(v_{\mathrm{Hi}} v_{\mathrm{Bo}}^{-1} \right)^2$.

If the contraction occurs against the gravitational force vector, the integral on the right-hand side remains unchanged, but the left-hand side is now less pleasant:

$$\int_0^{v_{\mathrm{Hi\text{-}Bo},g}} v \left[\frac{1}{1 - v v_{\mathrm{Hi}}^{-1} - \kappa_g} \right] dv = \frac{-v_{\mathrm{Hi}}^{-1} v_{\mathrm{Hi\text{-}Bo},g} + (\kappa_g - 1) \left[\log (1 - \kappa_g) + \log \left(1 - \kappa_g - v_{\mathrm{Hi}}^{-1} v_{\mathrm{Hi\text{-}Bo},g} \right) \right]}{\left[v_{\mathrm{Hi}}^{-1} \right]^2} \tag{S 7}$$

where $\kappa_g = mg \left(F_{max} G \right)^{-1}$ is the reduced parasitic energy. Combination of eqs. S 4 and S 7 and solving for $v_{\mathrm{Hi\text{-}Bo},g}$ yields:*

$$v_{\mathrm{Hi\text{-}Bo},g} = v_{\mathrm{Hi}} (1 - \kappa_g) \left[1 + \mathrm{W} \left(-\exp \left(-1 - \frac{1}{2\Gamma} \frac{1}{1 - \kappa_g} \right) \right) \right] \tag{S 8}$$

Eqs. S 6 and S 8 are identical apart from two terms, $1 - \kappa_g$, an expression which directly reflects the truncation of the strain rate accessible to muscle, and the extent to which muscle work flows into gravitational potential versus kinetic energy, respectively (Labonte, 2023).

*This solution is valid if the involved parameters are positive and real.

An overview of the dimensionless quantities used in the main manuscript is provided in table Supplementary table 1.

Table Supplementary table 1 | Definition of relevant dimensionless quantities. m is body mass; v is centre-of-mass speed; g is the gravitational acceleration; ΔL is a characteristic displacement; k is a characteristic spring constant; W_ρ is the work density, i. e., the maximum work output per unit muscle mass; m_f is the ratio between muscle and body mass; G is a characteristic gear ratio; l_m is a characteristic fascicle length; $\dot{\varepsilon}_{max}$ is the maximum muscle strain rate; F_{max} is the maximum muscle force. See also Table 1 in the main manuscript.

Name	Definition	Interpretation
Froude-number	$\mathrm{Fr}^2 = mv^2(g\Delta L)^{-1}$	Ratio of kinetic and gravitational potential energy.
Strouhal-number	$\mathrm{St}^2 = mv^2(k\delta L^2)^{-1}$	Ratio of kinetic and elastic strain energy.
Borelli-number	$\mathrm{Bo}^2 = v^2(W_\rho m_f)^{-1}$	Ratio of kinetic energy and muscle work capacity.
Hill-number	$\mathrm{Hi}^2 = (Gv)^2(l_m\dot{\varepsilon}_{max})^{-2}$	Ratio of kinetic energy and muscle kinetic energy capacity
Physiological similarity index	$\Gamma = \mathrm{Bo}^2/\mathrm{Hi}^2$	Ratio of muscle kinetic energy and work capacity
Reduced parasitic energy	$\kappa_g = mg(F_{max}G)^{-1}$	Ratio of gravitational potential energy and muscle work capacity

Supplementary Note 2

A classic application of dynamic similarity indices is to estimate equivalent speeds that allow comparison of locomotor traits across sizes. As an illustrative example, consider gait transitions, which appear to occur at critical speeds that vary systematically with size (Heglund and Taylor, 1988; Heglund et al., 1974). We analysed the variation of the speed at the trot-gallop transition across sixteen mammals varying in body mass between 0.03-680 kg, measured by Heglund and Taylor (1988); Heglund et al. (1974). A simple power-law fit in log-log space yields $v_{TG} = 0.19m^{0.21}$. Note well that we included the data point for a 680 kg horse from Heglund et al. (1974) in this regression, which appears to have been excluded in later work for unspecified reasons. For animals with a body mass of 10 mg and 40 t, this relationship predicts extrapolated transition speeds of 0.135 and 14.5 m s^{-1}, respectively (Figure Supplementary figure 1 b). It is common practice to extrapolate equivalent speeds instead by assuming equal Froude numbers, $v \propto m^{1/6}$. To estimate the transition speed via the Froude number, we fit a linear model with fixed slope of 1/6 but variable intercept in log-log space, yielding $v_{TG} = 1.75m^{1/6}$m s^{-1} kg$^{-1/6}$. For a body masses of 10 mg and 40 t, this predicts transition speeds of 0.26 and 10 m s^{-1}, respectively. Last, we extrapolate speeds by fitting directly the Hill-Borelli-number. To this end, we use the parameters from Table 1 in the main manuscript, and leave the intercept free as for the Froude number. This procedure is mathematically equivalent to fitting a different value for η, which then changes physical interpretation: η is now related to the fraction of maximum possible performance that is realised; the fit yielded $\eta = 0.46$. For masses of 10 mg and 40 t, this procedure predicts transition speeds of 0.04 and 0 m s^{-1}, respectively; the 40 t dinosaur has $\kappa_g > 1$ and thus would be unable to move. With some dinosaur species likely weighing more than 40 t (e. g., Carballido et al., 2017), this suggests that some extinct giants may have departed from expected allometric relationships by evolving ‘unique’ muscular properties (e. g., atypical fascicle lengths or muscle architecture, modified histochemistry). Last, we extract a linearised slope in log-log space for the prediction via the Hill-Borelli number: we calculate the speed for all 22 points in the data set via the least-squares fit conducted above, and then conduct an ordinary least-squares regression on the predictions. This procedure yields $v_{TG} = 0.22m^{0.18}$, close to the power law fit, and so demonstrating that predictions from the Hill-Borelli number, the Froude number, and a simple power law are hard to distinguish in this region without accurate and densely sampled data.

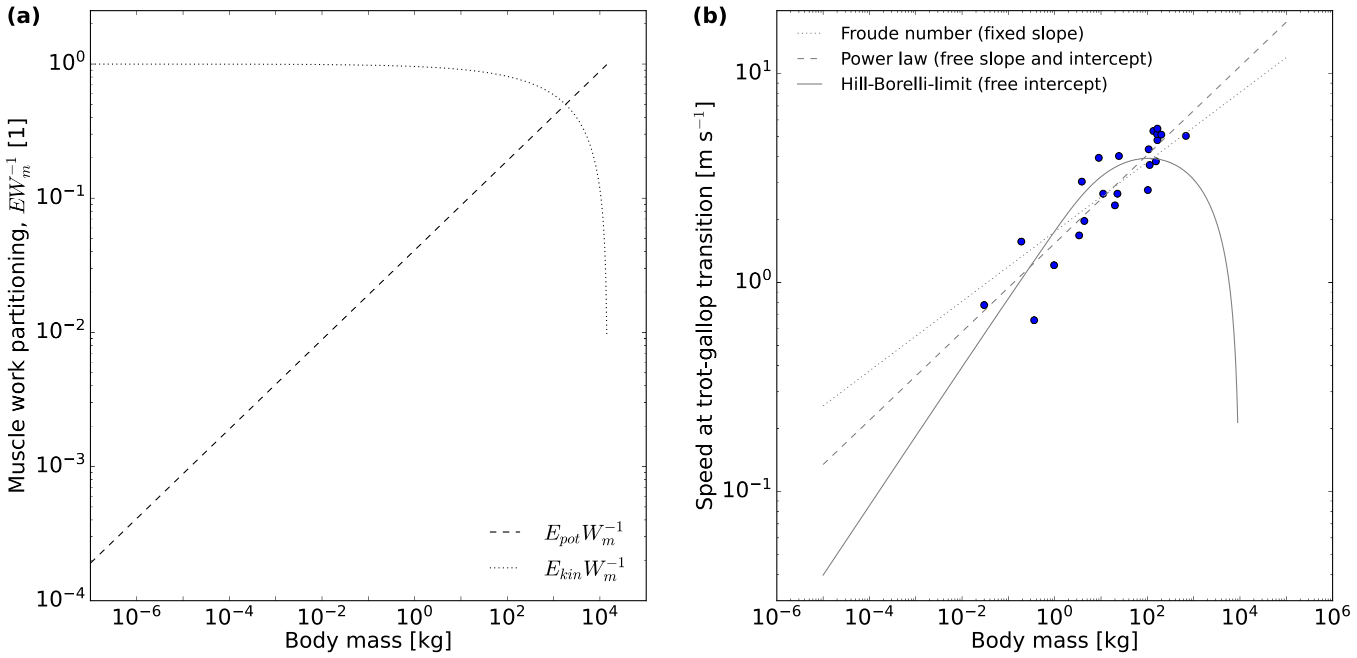

Figure Supplementary figure 1 | **(a)** Geometric similarity predicts a change in the ratio of weight force to maximal ground reaction force, $\kappa_g = mg(F_{\max}G)^{-1} \approx 0.041m^{1/3}\,\text{kg}^{-1/3}$ (see Table 1 in the main manuscript). A fraction κ_g of every unit of muscle work flows into gravitational potential energy, leaving a smaller fraction $1-\kappa_g$ to flow into kinetic energy. Because κ_g grows with size, larger animals thus have to invest a larger share of their muscle work into gravitational potential energy. However, even for an animal with a mass of 10 kg, gravitational potential energy costs during stance consume a mere 10% of the muscle work, $\kappa_g \approx 0.1$, and only for animals heavier than about 2 t does κ_g exceed 0.5; the majority of terrestrial animals may thus be considered gravitationally indifferent. **(b)** Dynamic similarity indices can be used to define characteristic speeds across sizes. In seminal work, Heglund and others showed that the speed at which mammalian tetrapods transition from a trot to gallop varies in a regular fashion with size, as indicated by a simple power law fit (dashed line. Heglund and Taylor, 1988; Heglund et al., 1974). The slope of this fit is close to the slope predicted by the Froude number, the dynamic similarity index used almost exclusively in comparative work on extant and extinct animals (dotted line, fitted as an ordinary least squares regression with fixed slope of 1/6 and free intercept). The Hill-Borelli-limit, as defined by eq. 5 in the main manuscript, predicts a similar scaling of the transition speed in this region, but differs meaningfully from monotonous power-law predictions when extrapolated to smaller and larger animals.

Supplementary Discussion 1

Hirt et al. (2017) proposed a model to explain the ‘hump-shape’ relationship between maximum running speed and animal size, and demonstrated exciting agreement between this model and data on terrestrial, aerial and aquatic animals. The premise of the model may be summarised as follows: larger animals can in general move faster, but it also takes them longer to accelerate to their top speed. This increase in acceleration time brings an inevitable increase in energy demand. Above some critical mass, the provision of energy can no longer keep up with the demand — absolute speed decreases.

Mathematically, Hirt et al. described this concept as the competition between two terms:

$$v_{max} = am^{\mu_1}\left(1 - exp(-bm^{\mu_2})\right) \tag{S 9}$$

where $a, b > 0$ are dimensionless constants (but see below), m is the body mass, and μ_1 and μ_2 are scaling coefficients related to the power-law relationships of variables y_i with respect to mass, $y_i \propto m^{\mu_i}$, the meaning of which will be discussed shortly. Can this model explain a ‘hump-shaped’ relationship between v_{max} and

mass m?

A 'hump-shape' implies the existence of a local maximum at a positive, real value of m. The existence and location of extrema may be evaluated by inspection of the root of the derivative of eq. S 9 with respect to mass:

$$m = \sqrt[\mu_2]{\left[\frac{\mu_1 - \mu_2 W\left[\exp\left(\frac{\mu_1}{\mu_2}\right)\frac{\mu_1}{\mu_2}\right]}{b\mu_2}\right]} \tag{S 10}$$

where W is the Lambert W-function. If the variables μ_i are both real and positive, the only real solution of the term bracketed by the Lambert W-function is μ_1/μ_2, so that there is only one extremum, located at $m = 0$. Hence, $\text{sign}(\mu_1) \neq \text{sign}(\mu_2)$ is a necessary condition for the existence of a "hump" – one of the two parameters must be negative. Although this is mathematically possible, it is unclear whether it would also be consistent with the conceptual interpretation of the variables μ_1 and μ_2 — a negative μ_i may well be physiologically or physically implausible.

The only specified numerical expectation for μ_1, which is introduced to describe the relationship between the theoretical maximum speed and body mass, $v_{max,t} \propto m^{\mu_1}$, is $\mu_1 > 0$ (Hirt et al., 2017), i. e. that speed generally increases with size. This assumption is plausible and consistent with the general biomechanical scaling literature. Having established the physical restriction $a, b, \mu_1 > 0$, it follows that a 'hump-shaped' relationship between mass and speed requires $\mu_2 < 0$, which is indeed consistent with the fitted estimate provided in Table S 4 of the supplement to the original publication, but in noteworthy contrast to the physically and empirically grounded estimate of $\mu_2 > 0.51$ that is provided in the main text (for terrestrial animals, $a = 142$, $b = 2.4$, $\mu_1 = 0.24$, $\mu_2 = -0.72$; the numbers for aquatic and flying animals are similar, see the supplemental material in Hirt et al., 2017). Hirt et al. (2017) did not discuss this difference between the estimate and the fit — is a negative value of μ_2 plausible?

The coefficient μ_2 is introduced in an empirical relationship between attained speed and mass (Hirt et al., 2017):

$$v(t) = v_{max,t}\left[1 - \exp(-k\tau)\right] = v_{max,t}\left[1 - \exp(-m^{\mu_2})\right] \tag{S 11}$$

The parameter k is labelled an 'acceleration constant', and τ is a 'critical time available for maximum acceleration' (Hirt et al., 2017). In order to assess the biologically plausible range for μ_2, we may follow Hirt et al. and write:

$$k\tau \propto m^{\mu_3} m^{\mu_4} \propto m^{\mu_3 + \mu_4} \propto m^{\mu_2} \tag{S 12}$$

Based on the assumption that τ is proportional to muscle volume (it is related to energy storage capacity according to Hirt et al., 2017), $\mu_4 \approx 1$, i. e. the fraction of body mass specialised as muscle tissue is constant with mass, consistent with empirical data (Bishop et al., 2021). $\mu_2 < 0$ thus requires $\mu_3 < -1$. The form of eq. S 11 implies that k is a decay constant of dimension $[\mathrm{t}^{-1}]$, but it is instead treated as an acceleration of dimension $[\mathrm{L\,t}^{-2}]$ (Hirt et al., 2017). This interpretation introduces dimensional inconsistency: eq. S 11 is missing a characteristic speed. This omission can be resolved in one of two ways.

First, we may follow the authors' implicit assumption that the characteristic speed required to ensure dimensional consistency is independent of mass, so that k is indeed solely proportional to an acceleration. From Newton's second law, it then follows that $k \propto F/m \propto m^{\mu_3}$, where F is the maximum available force; assuming isometry, $F \propto m^{2/3}$ so that $\mu_3 = -1/3$. Animals may well defy the default assumption of geometric similarity so that μ_3 may be somewhat smaller. However, a hump shape requires $\mu_3 < -1$, which would imply that the *absolute* maximum force animals can generate decreases with mass, which disagrees with abundant empirical data (Alexander, 1985), and is physiologically dubious (Alexander, 2003; Biewener and Patek, 2018). Second, and alternatively, one may assume that k is indeed a characteristic frequency, so that kt is a dimensionless ratio of two time scales. It is not immediately obvious what the biological interpretation

of k may be, but a plausible null hypothesis is that the system is described by one single time scale, in which case $\mu_3 = -\mu_4$, i. e. $k\tau$ is independent of mass. Both scenarios suggest $\mu_2 \geq 0$, consistent with the empirical estimates provided by Hirt et al in the text ($\mu_2 > 0.51$), but in contradiction with the fitted negative values. The prediction of a 'hump shape' with the model developed by Hirt et al unequivocally requires an unphysiological and unphysical choice of model parameters.

The fundamental logical problem with the 'supply-demand' model may also be illustrated in a simpler way: it implies that larger animals have relatively slower acceleration, and that the time τ over which they can accelerate before muscles fatigue changes with mass, so leading to a hump-shape. However, simple linear dynamics yield:

$$v_{max} = a\tau \propto m^{\mu_3+\mu_4} \tag{S 13}$$

which is monotonous regardless of the values of μ_3 and μ_4.

Although the model proposed by Hirt et al. reproduces the hump-shape observed in the empirical data, this mathematical result requires violation of the physical and physiological constraints imposed on the model parameters. The supply-demand argument as put forward by Hirt et al. fails to explain why the largest animals are not the fastest, and should not be considered a general scaling law (for a no less affirmative critique, see Günther et al., 2021).

References

Alexander, R. (1985). The maximum forces exerted by animals. *Journal of Experimental Biology* **115**, 231–238.

Alexander, R. M. (2003). *Principles of Animal Locomotion*. Princeton University Press.

Biewener, A. and Patek, S. (2018). *Animal locomotion*. Oxford University Press.

Bishop, P. J., Wright, M. A. and Pierce, S. E. (2021). Whole-limb scaling of muscle mass and force-generating capacity in amniotes. *PeerJ* **9**, e12574.

Carballido, J. L., Pol, D., Otero, A., Cerda, I. A., Salgado, L., Garrido, A. C., Ramezani, J., Cúneo, N. R. and Krause, J. M. (2017). A new giant titanosaur sheds light on body mass evolution among sauropod dinosaurs. *Proceedings of the Royal Society B: Biological Sciences* **284**, 20171219.

Günther, M., Rockenfeller, R., Weihmann, T., Haeufle, D. F. B., Götz, T. and Schmitt, S. (2021). Rules of nature's formula run: Muscle mechanics during late stance is the key to explaining maximum running speed. *Journal of Theoretical Biology* **523**, 110714.

Heglund, N. C. and Taylor, C. R. (1988). Speed, stride frequency and energy cost per stride: how do they change with body size and gait? *Journal of Experimental Biology* **138**, 301–318.

Heglund, N. C., Taylor, C. R. and McMahon, T. A. (1974). Scaling stride frequency and gait to animal size: Mice to horses. *Science* **186**, 1112–1113.

Hirt, M. R., Jetz, W., Rall, B. C. and Brose, U. (2017). A general scaling law reveals why the largest animals are not the fastest. *Nature Ecology & Evolution* **1**, 1116–1122.

Labonte, D. (2023). A theory of physiological similarity for muscle-driven motion. *PNAS* **120**, e2221217120.

McMahon, T. A. (1984). *Muscles, reflexes, and locomotion*, volume 10. Princeton University Press.
